# Peripheral blood mononuclear cell gene expression signatures predict long-term survivorship in canine DLBCL

**DOI:** 10.1038/s41598-026-44677-0

**Published:** 2026-03-17

**Authors:** Kirthana Rao, Zechuan Rao, Angelina Huang, Scott Heston, Max Wang, Ümmügülsüm Yildiz-Altay, Fatima Qutab, Danny A. Kwong, Heather L. Gardner, Jillian M. Richmond, Cheryl A. London

**Affiliations:** 1 Department of Dermatology, UMass Chan Medical School, Worcester, MA USA; 2 Morehouse School of Medicine, Atlanta, GA USA; 3 Texas A&M University College of Medicine, Bryan, TX USA; 4 Tufts Cummings School of Veterinary Medicine, North Grafton, MA USA; 5 Present Address: Yale University School of Medicine, New Haven, CT USA

**Keywords:** Diffuse large B cell lymphoma (DLBCL), Canine (dog), Cancer immunotherapy, Interferon (IFN), Liquid/blood biopsy, Immune landscape, Biomarkers, Cancer, Immunology, Oncology

## Abstract

Pet dogs spontaneously develop a form of diffuse large B cell lymphoma (DLBCL) that recapitulates many of the features of double hit (*MYC/BCL2*) human DLBCL. We recently completed a clinical trial in dogs with DLBCL using a combination of canine anti-CD20 antibody and low dose doxorubicin followed by one of three small molecule immune-modulating agents (KPT-9274, TAK-981 or RV1001). Clinical outcomes and tumor specific biomarkers of response from these dogs have been previously reported. In this study, we used the NanoString Canine IO panel to assess dynamic changes in gene counts from peripheral blood mononuclear cells (PBMCs) collected longitudinally from these from dogs over the course of their treatment to identify immune correlates associated with early relapse versus long-term survivorship. Increases in interferon-stimulated gene (ISG) signatures and immune skewing genes [*CCR9*,* CD209* (DC-SIGN), *CMKLR* and *DDX58* (RIG-I)] were associated with shorter (< 400 day) survival times and early relapse. In contrast, *CD1E* and *CCL14* were elevated post-immunotherapy in long-term (> 400 day) survivors, suggesting that these may be associated with protective immune signatures. Examining genes that were expressed in short- versus long-term survivors early on in the treatment regimen identified *TBHD*,* NPNT* and *ISG20* as elevated in dogs with shorter survival times at day 7. To facilitate point-of-care PBMC gene expression testing that could be used to distinguish those dogs likely to require more intensive treatment regimens in advance of relapse, we developed qPCR assays for *TBHD*,* NPNT* and *ISG20*. Together these data provide proof of principle that biomarker interrogation in PBMCs can help predict early relapse and poor responders to inform clinical management of DLBCL.

## Introduction

Diffuse large B cell lymphoma (DLBCL) represents one of the most common forms of non-Hodgkin lymphoma in humans^1^. The current standard therapy for human DLBCL integrates B cell depletion (rituximab) with multi-agent chemotherapy (CHOP), which has significantly enhanced efficacy compared to protocols that contain only cytotoxic agents^2–4^. While this results in long-term survivorship for over 70% of affected patients, challenges remain to effectively treat those who fail to respond or experience early relapse. The intensive regimen is also associated with cardiotoxicity and may not be well tolerated by elderly patients^5–7^. Consequently, there have been recent efforts to develop treatment regimens that are less dose-intense, or incorporate small molecule inhibitor/immunotherapy combinations without the inclusion of cytotoxic agents^8–10^. However, no consistent biomarkers exist to predict early relapse in elderly/frail patients and as such, significant challenges remain with respect to identifying those patients likely to benefit from non-CHOP based regimens.

Dogs serve as a spontaneous large animal model of DLBCL^11^, and have been instrumental in testing of novel small molecule inhibitors such as ibrutinib^12^ and acalabrutinib^13^, as well as chimeric antigen receptor (CAR) T cell based therapies^14^. We leveraged DLBCL in pet dogs as a spontaneous large animal model to determine whether a modified low dose chemo-immunotherapy regimen could achieve outcomes similar to CHOP without the associated toxicities^15–17^. Specifically, we treated dogs with three different protocols including a caninized anti-CD20 antibody (rituximab equivalent), low dose doxorubicin and one of 3 small molecule immune modulating drugs (TAK981, KPT9274 and RV1001^15,16^). We showed that all three regimens were extremely well tolerated, resulting in median survival times that were equivalent or superior to those achieved with CHOP alone^15,16^. However, within each arm there were dogs that did not respond well to the therapy, experiencing early relapse and short survival times. In the current study, we utilized longitudinal collections of peripheral blood mononuclear cells (PBMCs) from dogs treated in this study to understand whether long-term survivorship could be predicted based on the immune landscape as assessed by PBMC gene expression signatures. Ultimately, such a non-invasive approach has the potential to provide clinical meaningful information relevant for informing treatment strategies in both dogs and humans.

## Materials & methods

### Study design

**Fig. 1:**
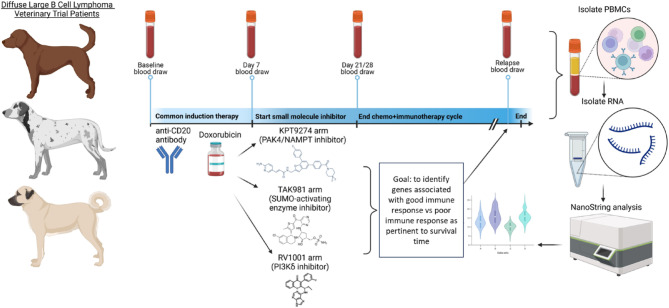
Study overview. Dogs analyzed in this study all received anti-CD20 antibody and doxorubicin induction therapy and were separated into 3 treatment arms with small molecule inhibitors TAK-981, KPT-9274 and RV1001. Blood was drawn and analyzed at baseline (time A), post-B cell depletion (day 7, time B), post-completion of the first chemoimmunotherapy cycle (time 21-28d depending on the immunotherapy drug/regimen, time C) and at relapse (time D). PBMCs were isolated and RNA was extracted for NanoString analysis with the goal of identifying peripheral biomarkers associated with long-term survival.

This study utilized PBMCs from pet dogs enrolled in a non-randomized clinical trial aimed at evaluating the effectiveness of combining a canine anti-CD20 monoclonal antibody with doxorubicin and specific targeted small molecule inhibitors (RV1001, KPT-9274, TAK-981) for the treatment of diffuse large B-cell lymphoma (DLBCL). The trial was conducted following protocol #G2017-110, approved by the Tufts University Institutional Animal Care and Use Committee (IACUC), and studies were conducted in accordance with the National Institutes of Health (NIH) Guide for the Care and Use of Laboratory Animals. Informed consent was obtained from all participating dog owners; all potential risks involved in the study were discussed with owners prior to enrollment. The protocol and outcomes have been previously described^15,16^; an overview of the study is provided in Fig. 1. Briefly, KPT-9274 is a dual inhibitor of PAK4 (p21-activated kinase 4) and NAMPT; PAK4 is a serine/threonine protein kinase that promotes tumor cell survival and proliferation, and NAMPT is a critical enzyme for NAD+ salvage pathway synthesis^18,19^. TAK-981 is a SUMO-activating enzyme inhibitor and induces genes associated with Type I interferon responses^20^. RV1001 is a PI3Kδ inhibitor, a pathway known to be enriched in human and canine DLBCL^21^. Each dog received a combination treatment regimen comprising doxorubicin, anti-CD20 antibodies, and a small molecule inhibitor specific to their study group (Table 1).

Blood collection was performed at every study visit, using CPT tubes (BD Biosciences) for PBMC purification^22^. PBMCs were isolated from the CPT tubes according to the manufacturer’s instructions and cryopreserved for RNA isolation and downstream analyses. For this study, we selected 4 timepoints for analysis: baseline (time 0, denoted as time A), post-B cell depletion (time 7d, denoted as time B), post-completion of the first chemoimmunotherapy cycle (time 21-28d depending on the immunotherapy drug/regimen, denoted as time C) and at relapse (denoted as time D). We confirm the study design is reported in accordance with ARRIVE guidelines.

**Table 1:** Study arms from DLBCL veterinary trial.

Treatment arm	Long-term survivors- Yes (LTS-Y) N; median days survived *±* standard deviation	Long-term survivors - No (LTS-*N*) N; median days survived *±* standard deviation
TAK981	2; 612 *±* 288	3; 292 *±* 124
KPT9274	4; 688 *±* 119	3; 62 *±* 50
RV1001	4; 751 *±* 146	2; 55 *±* 52

### Inclusion & exclusion criteria

Dogs were eligible for inclusion if they met the following criteria: a diagnosis of DLBCL established via lymph node biopsy, a minimum weight of 10 kg, an age of at least one year, the presence of at least two peripheral lymph nodes measuring 2 cm or greater in diameter, and adequate organ function confirmed through standard laboratory assessments. Dogs were excluded if they presented with uncontrolled autoimmune conditions, severe cardiovascular disease, recent major surgery, pregnancy, lactation, central nervous system involvement, or were receiving concurrent medications that could potentially confound study outcomes.

### RNA isolation & NanoString analysis

RNA from canine PBMCs collected at Days 0 (pre-treatment), D7, D21/28 (depending on treatment arm) and at relapse were extracted using QIAGEN RNeasy mini kits with gDNA eliminator tubes per the manufacturer’s protocol. RNA concentrations were quantified using a Nanodrop spectrophotometer. The nCounter Canine IO Panel was employed for hybridization, using 100ng of RNA (MAX machine) per sample incubated over a 19.5-hour period. Microarrays were analyzed on the NanoString nCounter Sprint system according to the manufacturer’s guidelines.

### Microarray data & statistical analysis

Data analysis was conducted using nSolver software and NanoString partner analysis ROSALIND^®^ (https://rosalind.bio/*)* nCounter software. Housekeeping probes for normalization were chosen by analyzing common selected housekeeping genes across all 3 treatment arms using nSolver software. These genes were then selected, and normalized count files were generated in nSolver and imported into ROSALIND software. Comparisons across arms with correction for time were performed in ROSALIND, and two-way comparisons for good versus poor outcomes were performed in nSolver software using a progression free survival time (PFS) > 90 days (coinciding with completion of the post-completion of the first chemoimmunotherapy cycle) and/or overall survival time (OST) of > 400 days which is more than double the reported median survival time for single agent doxorubicin treatment^23–25^. Significant genes were chosen as log2fold change less than or greater than 1.5, and *p* < 0.05. To help ensure stringency in biomarker identification, we also incorporated P-value adjustment was performed using the Benjamini-Hochberg method. Data were graphed in GraphPad Prism software version 10 or newer, and two-way ANOVAs for long-term survivorship and time were analyzed with Dunnett’s post hoc tests comparing to baseline time point.

### Realtime PCR & statistical analysis

RNA from canine PBMCs was isolated using RNeasy kits (Qiagen) in accordance with the manufacturer’s instructions. Complementary DNA (cDNA) synthesis was performed using iScript kits (BioRad). Quantitative PCR was carried out using SYBR green kits (BioRad) on a CFX96 instrument (BioRad), running 40 cycles at 56 °C. Primer sequences are listed in Table 2. The ΔΔCT method was used for calculating copy numbers in Microsoft Excel. Data were graphed in GraphPad prism software and student’s t tests were used to compare long-term survivorship yes versus no groups.

**Table 2:** qPCR primers used for this study.

Gene name	Forward primer	Reverse primer
*THBD*	CTC GTG CCA TAA ACT GTG CG	ACT CAC ACT TGC TCC CGA AG
*NPNT*	GAA GCC TCG GCC CTG CAA G	AGC ATA TAT CCG TTG AGA CAG TA
*ISG20*	CCC GCT GCA GCC TCG TGG A	TGG GTC CTG TAG TCA GTG ATC TC
*GAPDH*	GAT GGG CGT GAA CCA TGA G	TCA TGA GGC CCT CCA CGA T

## Results

**Fig. 2:**
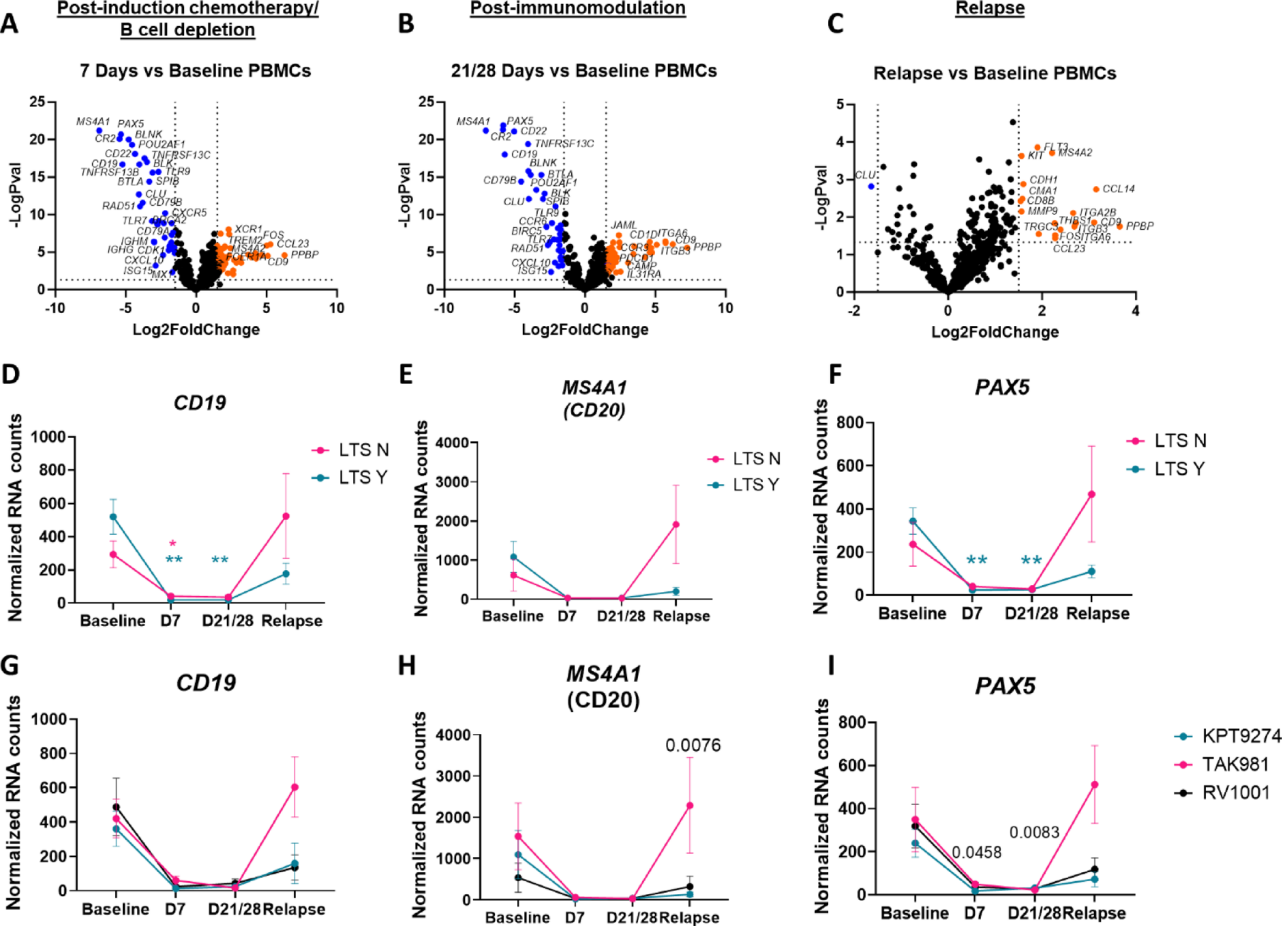
Examination of PBMC gene signatures in DLBCL trial patients across all arms confirms loss of B cell signatures. (**A**) Post-B cell depletion, (**B**) post-completion of the first chemoimmunotherapy cycle and (**C**) relapse gene expression signatures compared to baseline. Comparison of B cell gene signatures in long-term survivors (LTS) yes versus no reveals similar levels of peripheral B cell depletion as assessed by (**D**) CD19, (**E**) MS4A1 (CD20) and (**F**) PAX5 expression. *n* = 10 LTS Y, *n* = 8 LTS N. (**G**) CD19, (**H**) MS4A1 (CD20) and (**I**) PAX5 expression by treatment arm. *n* = 5 TAK-981, 7 KPT-9274 and 6 RV1001 recipients. (two-way ANOVA with Dunnet’s post hoc tests significant as indicated).

The primary goal of this work was to determine whether gene expression signatures in PBMCs could predict long-term survivorship (defined as overall survival time greater than 400 days) across the three treatment cohorts (Fig. 1). With respect to progression free survival (PFS), there were roughly equal numbers of dogs with long (*n* = 10) and short (*n* = 8) PFS (defined as *≤* 90 days or > 90 days, respectively based on our prior study endpoints^16)^. Importantly, there was an equal distribution of dogs with short and long term PFS in each of the three cohorts (Table 1). Using the NanoString Canine IO Panel, we evaluated PBMC gene expression signatures across all dogs at 4 key time points: baseline (time 0, time point A), post-B cell depletion (time 7d, time point B), post-immunotherapy (time 21-28d depending on the immunotherapy drug/regimen, time point C) and at relapse (time point D). Loss of B cell gene signatures (*PAX5*,* MS4A1*,* CD19*) was noted in nearly all dogs at D7 and D21/28 (Fig. 2A–C), concordant with B cell depletion secondary to use of the canine anti-CD20 mAb^15^. At relapse, the B cell signatures recovered (Fig. 2D–F), supporting the loss of clinical remission. Importantly, the kinetics of B cell specific gene expression signatures were consistent across the three different treatment regimens (Fig. 2G–I).

**Fig. 3:**
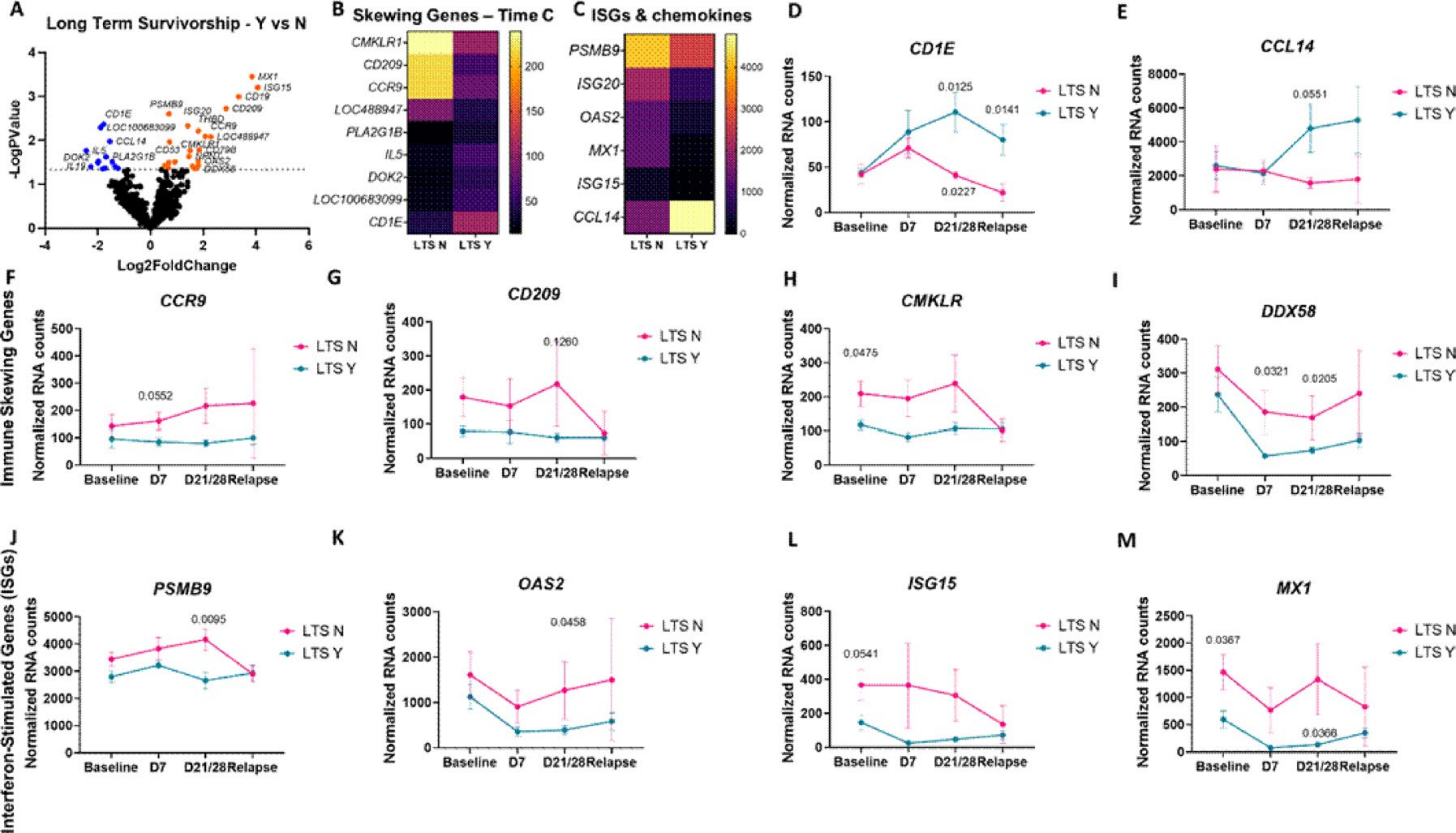
Examining PBMC gene expression by survivorship following chemoimmunotherapy regimen (time C) identifies immune skewing genes and interferon-stimulated genes (ISGs) are associated with lower survival time, whereas CD1E and CCL14 are associated with long-term survivorship. (**A**) Unbiased gene expression analysis in long-term survivors yes versus no across all time points (*n* = 10 LTS Y, 8 LTS N). (**B**) Heatmap of immune skewing genes and (**C**) ISGs and chemokines at time point C, which represents the end of the treatment regimen, by survivorship. (**D**) CD1E and (**E**) CCL14 counts over time have a statistically significant increase post-immunotherapy (time point C) in long-term survivors. Immune skewing genes including (**F**) CCR9, (**G**) CD209, (**H**) CMKLR and (**I**) DDX58 were elevated or trending higher in dogs with shorter overall survival times. ISGs including (**J**) PSMB9, (**K**) OAS2, (**L**) ISG15 and (**M**) MX1 are increased in poor responders compared to long-term survivors. (two-way ANOVA with Dunnet’s post hoc tests significant as indicated).

To understand potential gene expression biomarkers that could help guide treatment decisions, we classified dogs as good responders if they had a progression free survival (PFS) > 90 days which corresponded to completion of the chemo-immunotherapy protocol and/or if they had an overall survival time (OST) > 400 days. Unbiased gene expression analysis of PBMCs from all dogs at all time points, examining long versus short term survivorship, revealed several differentially expressed genes (DEGs) that could serve as candidate biomarkers (Fig. 3A–C). Next, we compared expression of these DEGs singly over time. *CD1E* and *CCL14* predicted long-term survivorship post-chemo-immunotherapy (Fig. 3D,E). DEGs that mediate immune skewing were differentially associated with survivorship. Specifically, *CCR9*, which is associated with mucosal homing T cells, was increased in dogs with short survival (Fig. 3F). *CD209*, which is a marker of M2 macrophages, was also elevated in those with poor outcomes (Fig. 3G). Two additional genes *CMKLR*, an atypical chemokine receptor, and *DDX58*, a nucleic acid sensor, were also elevated in dogs with poor survival across all time points (Fig. 3H,I). Interestingly, interferon stimulated genes (ISGs) including *PSMB9*,* OAS2*,* ISG15* and *MX1*, were associated with a worse outcome at all time points (Fig. 3J–M).

**Fig. 4:**
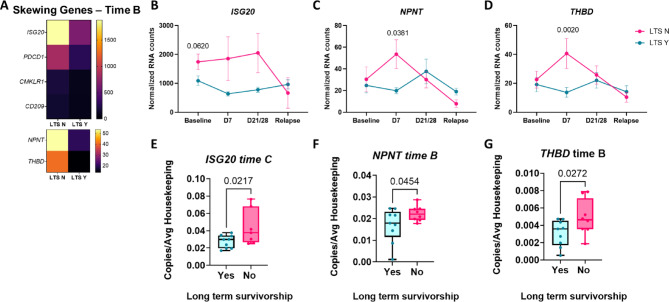
Identifying genes associated with poor survivorship at time points B (post-B cell depletion) and C (post chemoimmunotherapy) for catching patients who need more aggressive treatment. (**A**) Heatmap of key DEGs at time point B, which represents day 7 post-B cell depletion. (**B**) ISG20, (**C**) NPNT and (**D**) THBD counts over time. (**E**) ISG20, (**F**) NPNT and (**G**) THBD qPCR validation at the indicated time points. (two-way ANOVA with Dunnet’s post hoc tests significant as indicated; unpaired t tests significant as indicated; ISG20 did not reach statistical significance at time B in PBMC qPCR assays).

Next we specifically examined genes that could be used to predict outcome at early time points post treatment initiation (time B, D7 and time C, D21/28) as these mark a critical point during treatment initiation that defines depletion of B cells and induction of remission, respectively (Fig. 4A). Elevated ISGs such as *ISG20* predicted poor survivorship at baseline, D7 and D21/28 post-treatment in the microarray (Fig. 4B). Elevated *NPNT*, or the nephronectin gene, and *THBD*, which encodes thrombomodulin, and predicted poor survivorship at time B (Fig. 4C,D). To validate these findings, we used qPCR to confirm that *ISG20*,* NPNT* and *THBD* expression were significantly elevated in dogs with worse outcomes (Fig. 4E–G). *ISG20* reached statistical significance in qPCR assays performed on samples obtained at time C, whereas *NPNT* and *THBD* reached significance in assays for time B.

## Discussion

Over the past decade there has been rapid development of several non-invasive methodologies capable of predicting outcomes and/or directing therapy in cancer patients. Collectively known as liquid biopsy, this typically involves serial blood collection to analyze circulating elements (i.e., DNA, RNA, exosomes) released by tumor cells. We reasoned that a similar approach could be used in the context of immunotherapy studies in which PBMC gene signatures could serve as a surrogate biomarker associated with treatment response and patient outcomes. We leveraged a previous study in dogs with DLBCL in which they were randomized to receive one of three different chemo-immunotherapy regimens^15,16^ and analyzed gene expression signatures at various time points over the course of therapy. Using the NanoString canine IO panel, we found that the expression of two genes, *CD1E* and *CCL14*, represent potential biomarkers of long-term survivorship post-treatment.

CD1E is a member of the immunoglobulin supergene family which delivers lipid and glycolipid antigens to T cells^26^. Several studies have evaluated the preclinical efficacy of CD1 restricted T cell responses in anti-tumor immunity^27,28^. Harnessing CD1 restricted responses may therefore represent a novel immunotherapy based approach for canine DLBCL. CCL14 is a small cytokine within the CC chemokine family. It is expressed in several tissues including the spleen, bone marrow, liver, muscle, and gut, promoting the activation and migration of immune cells. Because CCL14 can recruit tumor infiltrating immune cells (TIIC) such as CD4 + and CD8 + T cells, neutrophils, and B cells, it has been associated with amplifying anti-tumor immunity in hepatocellular carcinoma^29^ and lung adenocarcinoma^30^. However, CCL14 has a negative association with prognosis in gastric^31^ and thyroid cancer^32^. The precise roles of CD1E and CCL14 in DLBCL have not yet been explored as most of the prior studies have been in the context of solid tumors.

We also identified immune skewing genes linked to a poor outcome across all time points assessed, including *CCR9*,* CD209*, *CMKLR* and *DDX58*. Typically associated with mucosal-homing T cells, CCR9 is a chemokine receptor that binds the ligand CCL25. CCR9 is also linked to less activated and less differentiated dendritic cell (DC) populations^33^ that can induce Tregs^34^. Interestingly, CCR9 expression in human DLBCL is highly correlated with gut involvement^35^. Given that we demonstrated robust B cell depletion in all dogs, however, it is more likely that *CCR9* is associated with poor T cell/DC responses to DLBCL in the context of the canine clinical trial. Another gene associated with poor immune skewing is *CD209* (also called DC-SIGN). In human DLBCL, malignant B cells recruit monocytes and DCs to support tumor cell survival^36^. Interestingly, a similar situation has been described in human peripheral T cell lymphoma in which lymphoma associated macrophages are intimately linked to disease progression^37^. CMKLR is a G-protein coupled receptor with homology to many other chemokine receptors^38^. Its role in cancer is controversial, with some studies supporting an anti-tumor role and others a pro-tumorigenic role (reviewed in^39^). *DDX58* encodes RIG-I and acts as an immune response factor and mediator of IFN antiviral responses. RIG-1 can promote protective anti-tumor immune responses in colon cancer^40^, and it can prevent breast cancer metastasis^41^, possibly through its ability to upregulate type 1 IFN as well as other cytokines^42^. It has been shown that lenalidomide, an agent now used to treat DLBCL in elderly/frail patients, acts in part by activating RIG-1 domains to inhibit tumor cell proliferation^43^. Consequently, it is not yet clear why upregulation of *DDX58* would be linked to poor outcomes in the context of chemo-immunotherapy.

Interestingly, we found that interferon-stimulated gene (ISG) signatures were associated with poor prognosis in this study. While historically it was believed that IFN responses were integral to anti-tumor immunity, it has become increasingly clear that they are highly contextual and can actually be pro-tumorigenic in specific tumor settings^44^. For example, in glioblastoma, IFN signatures are associated with a worse prognosis^45^. Additionally, in breast cancer, IFN signatures are associated with metastatic disease in specific tumor phenotypes: *ESR1+/ERBB2 −* tumor metastasis is associated with IFN expression whereas *ERBB2* + is not^46^. Here we show that the ISGs *PSMB9*,* ISG15*,* ISG20*,* OAS2* and *MX1* and the *DDX58* sensor which promotes IFN expression are associated with shorter overall survival time in canine DLBCL, regardless of the immunotherapeutic regimen used.

In the current study we found that two genes, *TBHD* and *NPNT*, were elevated in the PBMCs from dogs with poor outcomes post-B cell depletion. *TBHD* encodes thrombomodulin (TM), an endothelial cell receptor that binds thrombin converting it from a procoagulant enzyme to an anti-coagulant. High levels of plasma TM have been linked to outcome across several different tumor types including those of embryonal, epithelial and lymphatic origin^47,48^. Its role in lymphoma has not been previously described, however, high levels of TM and von Willebrand factor were associated with a worse outcome in children with acute lymphoblastic leukemia^49^. NPNT, also known as nephronectin, is an extracellular matrix protein in the epidermal growth factor (EGF) family. NPNT plays a large role in cell mobility, structure, and signaling as it binds integrin α8β1. Previous studies showed that it is overexpressed in breast cancer tissue and is associated with metastasis and poor prognosis^50–52^. It is interesting to note that blood levels of NPNT are elevated in autoimmune experimental autoimmune encephalitis (EAE) mice and that it influences disease pathology by modulating the Th17/Treg balance^53^. Additionally, NPNT expression is upregulated in mouse models of acute and chronic hepatitis, supporting recruitment of CD4 + T cells into the liver^54^. Its potential role in DLBCL has not been previously described.

Lastly, we demonstrated that a subset of genes identified in the nCounter analyses, *TBHD*,* NPNT* and *ISG20*, could be used to predict response to outcome using qRT-PCR. These have potential use for the development of a point-of-care assay (i.e., digital droplet PCR) to help predict which dogs with DLBCL may require more intense follow-up or an altered treatment regimen, based on the change in gene expression between baseline (day 0) and day 7 of the treatment protocol.

Limitations of our study include sample size, which had 5–7 pet dogs per treatment arm, but in some cases only *n* = 2 in long or short term progression free survival groups based on treatment responses. We still believe that reporting these data are valuable given the potential impact on veterinary practice and the applicability of pet dogs as a spontaneous large animal model of human cancer. Another limitation of our study was an inability to perform qPCR validation for *CCL14* or *CD1E*, as the primers we designed were unable to produce usable products. Future work would include prospective studies designed to credential these biomarkers and thereby enable enhanced precision therapy.

In summary, data generated from this clinical trial show that biomarkers correlating with outcome in the context of novel chemo-immunotherapy treatments for DLBCL can be discerned from longitudinal analysis of PBMCs, providing a mechanism for rapid assessment of treatment efficacy and subsequent therapeutic intervention as needed.

## Data Availability

Study data are provided within the manuscript, and have been deposited on the Gene Expression Omnibus (GEO) Database under accession GSE302459 at [https:/www.ncbi.nlm.nih.gov/geo/query/acc.cgi? acc=GSE302459].

## Abbreviations

DLBCL: Diffuse large B cell lymphoma
DC: Dendritic cell
CMKLR: Chemerin Chemokine-like receptor
IFN: Interferon
ISG: Interferon stimulated gene
LTS: Long-term survivorship
NPNT: Nephronectin
THBD: Thrombomodulin
